# A 77 year old man with gangrenous cholecystitis and incidental findings of multiple bilobar liver lipomatosis

**DOI:** 10.11604/pamj.2020.37.31.23071

**Published:** 2020-09-08

**Authors:** Danilo Coco, Silvana Leanza

**Affiliations:** 1 Department of General Surgery, Ospedali Riuniti Marche Nord, Pesaro, Italy,; 2 Department of General Surgery, Carlo Urbani Hospital, Jesi, Ancona, Italy

**Keywords:** Old man, liver-disease, steato-hepatitis, Madelung’s disease

## Image in medicine

A 77-year-old man was evaluated at the hospital because of fever, vomiting and upper right quadrant pain. CT evaluation revealed acute cholecystitis with peri-cholecystic fluid. The patient was admitted in the operating room where an emergency laparoscopic cholecystectomy was performed. During laparoscopy an incidental finding of bilobar liver lipomatosis was found. Non-Alcoholic-Fatty-Liver-Disease (NAFLD) or Non-Alcoholic-Steato-Hepatitis (NASH) or Madelung's disease. A diagnosis was not made.

**Figure 1:**
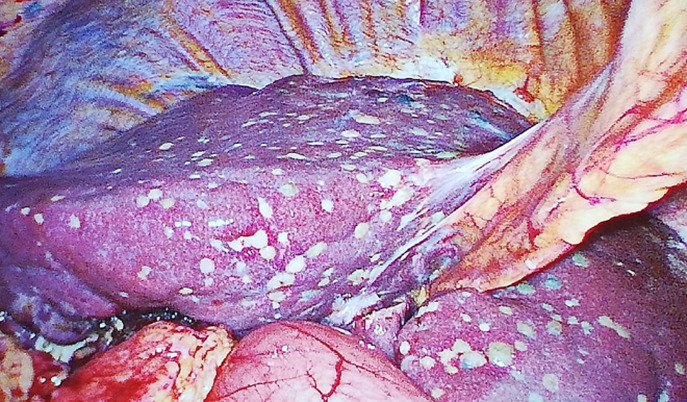
incidental findings of multiple bilobar liver lipomatosis during laparoscopic cholecystectomy

